# The utilization and delivery of safer smoking practices and services: a narrative synthesis of the literature

**DOI:** 10.1186/s12954-023-00875-x

**Published:** 2023-10-27

**Authors:** Abigail Tapper, Catherine Ahern, Zoe Graveline-Long, Noam G. Newberger, Jaclyn M. W. Hughto

**Affiliations:** 1 Boston, Massachusetts USA; 2 Department of Psychology, University of Rhode Island, Kingston, Rhode Island USA; 3 Department of Behavioral and Social Sciences, Brown University School of Public Health, Providence, RI 02903 USA; 4 Department of Epidemiology, Brown University School of Public Health, Providence, RI 02903 USA; 5 Center for Health Promotion and Health Equity, Brown University, Providence, RI 02903 USA; 6 The Fenway Institute, Fenway Health, Boston, MA 02215 USA

## Abstract

**Background:**

Providing sterile drug smoking materials to people who use drugs can prevent the acquisition of infectious diseases and reduce overdose risk. However, there is a lack of understanding of how these practices are being implemented and received by people who use drugs globally.

**Methods:**

A systematic review of safer smoking practices was conducted by searching PubMed, PsycInfo, Embase for relevant peer-reviewed, English-language publications from inception or the availability of online manuscripts through December 2022.

**Results:**

Overall, 32 peer-reviewed papers from six countries were included. 30 studies exclusively included people who use drugs as participants (*n* = 11 people who use drugs; generally, *n* = 17 people who smoke drugs, *n* = 2 people who inject drugs). One study included program staff serving people who use drugs, and one study included staff and people who use drugs. Sharing smoking equipment (e.g., pipes) was reported in 25 studies. People who use drugs in several studies reported that pipe sharing occurred for multiple reasons, including wanting to accumulate crack resin and protect themselves from social harms, such as police harassment. Across studies, smoking drugs, as opposed to injecting drugs, were described as a crucial method to reduce the risk of overdose, disease acquisition, and societal harms such as police violence. Ten studies found that when people who use drugs were provided with safer smoking materials, they engaged in fewer risky drug use behaviors (e.g., pipe sharing, using broken pipes) and showed improved health outcomes. However, participants across 11 studies reported barriers to accessing safer smoking services. Solutions to overcoming safer smoking access barriers were described in 17 studies and included utilizing peer workers and providing safer smoking materials to those who asked.

**Conclusion:**

This global review found that safer smoking practices are essential forms of harm reduction. International policies must be amended to help increase access to these essential tools. Additional research is also needed to evaluate the efficacy of and access to safer smoking services, particularly in the U.S. and other similar countries, where such practices are being implemented but have not been empirically studied in the literature.

## Introduction

Harm reduction is a collection of concepts and strategies that can be used to reduce adverse health consequences associated with drug use [1]. Harm reduction strategies can be conceptualized as a continuum of approaches from safer drug use practices to abstinence, with an underlying core ethos of a desire to meet people where they are at. As an alternative to the “zero tolerance” abstinence-only models of addiction treatment, the harm reduction model recognizes that abstinence may not be a desirable or achievable outcome for all people who use drugs [2]. Thus, practical strategies are necessary to reduce health-related harms associated with drug use (e.g., viral transmission of Human immunodeficiency virus (HIV) and Hepatitis C (HCV) through shared drug use equipment, fatal and nonfatal overdose), rather than exclusively targeting drug consumption itself [3–7].

Historically, harm reduction principles are actualized when individuals and groups take sometimes illegal measures to protect their communities. Once systemic structures recognize the value in these practices, they might become decriminalized and widely supported by public health institutions. As an example, supervised consumption sites have been created; these are spaces where individuals can use drugs in a sterile and monitored space with access to supplies and care. Legalized in certain European nations, Canada, and Australia, supervised consumption sites in the U.S. operated quietly and against the law [8]. With increased evaluations published globally, and within the country on unsanctioned supervised consumption sites [9], we see increased receptiveness in academic circles. In the U.S., this illicit practice of providing safe spaces to consume drugs recently gained popular ground with Rhode Island becoming the first state to legalize supervised consumption sites [10], and OnPoint in New York City opening the first SCS in the U.S. [11]. Other recent innovations in public health lifted up by the advocacy of people who use drugs include drug checking and safer smoking initiatives.

Harm reduction has traditionally focused on mitigating the risks of injection drug use (IDU) [7, 12–15] by providing access to sterile syringes via syringe service programs (SSPs) [16], and, more recently, supervised injection facilities [14, 17–20]. SSPs and the concept of risk reduction were adopted as public health strategies by several countries in the 1980s (e.g., Australia, Brazil, Denmark, Netherlands, some states in the U.S., United Kingdom) in the midst of the HIV/AIDS epidemic [7, 21]. In 1986, the World Health Organization was the first major international body to accept and endorse harm reduction [21], marking an influential shift in historically punitive global drug policies [22]. Other international bodies such as Joint United Nations Programme on HIV/AIDS, United Nations Office on Drugs and Crime, International Drug Policy Consortium, and United Nations Development Programme have joined in their endorsement of harm reduction [23].

Harm reduction services were originally focused on reducing adverse health outcomes for people injecting heroin [24]. Smoking drugs also carry health risks, including pulmonary distress [13, 25], COVID-19 [15], overdose (OD) [26], burns and lacerations on the lips [27, 28], tuberculosis [29], HIV, and HCV [3–5]. In order to mitigate these risks, some countries have led the way in developing safer smoking programs. Indeed, as early as the 1970s, informal drug consumption rooms, primarily inhalation-oriented spaces, were operating in the Netherlands [19, 20]. By 1999, Hamburg, Germany, operated 15 supervised inhalation spaces, and Switzerland introduced inhalation spaces by 2001 [19]. Similarly, in 2000, the Safer Crack Use Coalition of Toronto, Canada, began distributing ‘safer crack use kits’ to advocate for people who smoke drugs [9], a practice adopted by the Toronto city government in 2005 and recommended by Ontario, Canada in 2006 as ‘best practices’ for harm reduction programs [30].

Despite the increasing availability of safer smoking services internationally, harm reduction efforts targeting noninjection drug use have received comparatively less attention than those for IDU [15, 24, 27, 31, 32], even as health and social consequences associated with smoking substances are becoming better understood. People who smoke drugs are often characterized as a hard-to-reach population for social service programs [27] because these programs have traditionally been focused on the provision of supplies (e.g., syringes, naloxone) to people who inject drugs [33]. The distribution of safer smoking supplies (e.g., sterile pipes, stems, filters) by harm reduction organizations creates an opportunity to engage people who smoke drugs who may not otherwise access harm reduction programming [24, 30, 34]. Further, in 2019, the United Nations Office on Drugs and Crime called for the expansion of programs for people who use stimulants, particularly those providing safer smoking education and supplies [28].

The ongoing removal of drug policies that criminalize the provision of safer smoking materials in countries around the world [13, 24, 35], together with international calls for the expansion of safer smoking services [15, 21, 23, 31], has opened the door for the widespread implementation of these services in many regions. However, the extent to which safer smoking services are being provided globally is not well-understood. Moreover, synthesized data on access to and feasibility, acceptability, and efficacy of safer smoking harm reduction services are lacking in the literature. To close this research gap, we conducted a systematic review to summarize the available literature on (1) whether and how safer smoking interventions have been incorporated into harm reduction initiatives; (2) whether people who use drugs have access to safer smoking materials and services; (3) whether and how people who smoke drugs engage in safer smoking practices; and (4) the extent to which safer smoking practices and the availability of safer smoking services reduce the health-related risk of smoking drugs.

## Methods

The PRISMA reporting guidelines were used in the development of this protocol-driven report. The protocol was registered in PROSPERO: International Prospective Register of Systematic Reviews (ID: CRD42022345289).

### Inclusion and exclusion criteria

To be eligible for inclusion in this review, articles must have contained one or more of the following search terms from set A or B (see "Appendix"). Articles had to be written in English and published in a peer-reviewed journal as an original article. All articles were required to be based on studies involving human subjects. This review excluded other reviews, dissertations, conference abstracts and presentations, and commentaries, as well as studies that reported on harm reduction practices that did not explicitly discuss safer smoking services.

### Study identification

The authors generated a set of terms that aligned with the focus of the review (e.g., safer smoking, harm reduction). The first and second authors then consulted an expert librarian at Boston University, who helped design and conduct the electronic search strategy (See "Appendix"). To identify eligible studies, PubMed, PsycInfo, and Embase were searched from inception or the availability of online manuscripts through December 2022. Exact search terms for these databases were determined with preliminary inquiries and refined as needed. In PubMed, tiab (limiting to search terms to title or abstract) and mesh (medical subject headings) searches were implemented. A hand search of the bibliographies of retrieved articles was also conducted.

**Fig. 1:**
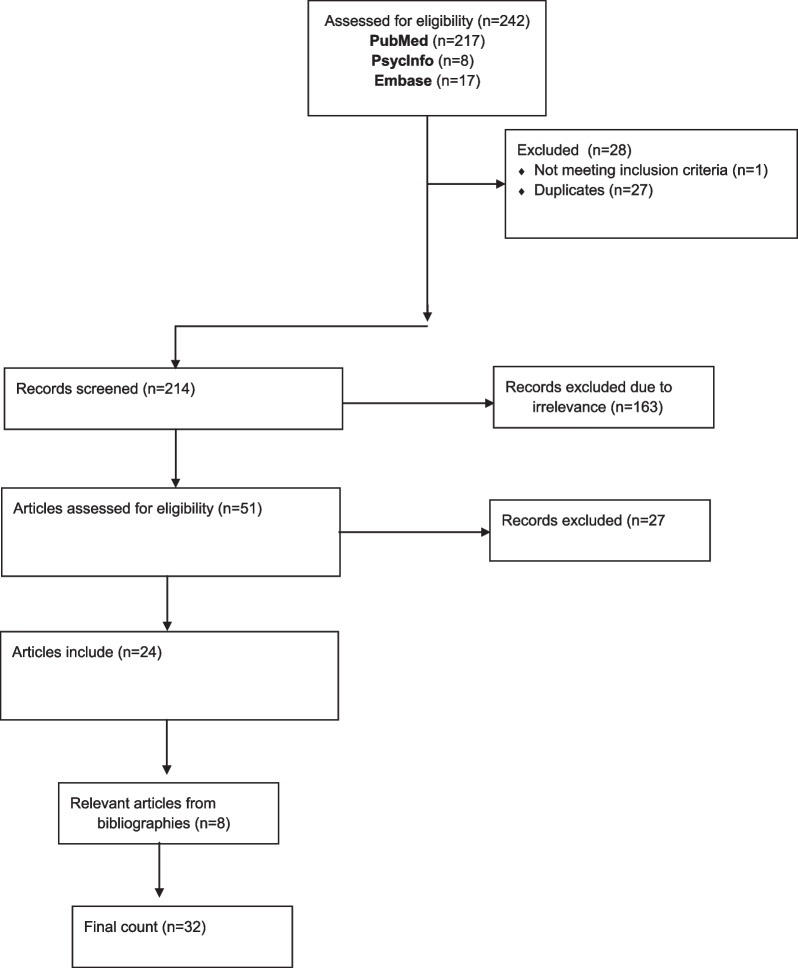
Review consort table

The initial search returned 214 articles. The first and second authors (AT and CA) examined abstracts and titles from the initial search to identify studies that appeared to meet the inclusion criteria. The full article was then obtained for all studies appearing to meet inclusion criteria or in instances where there was insufficient information from the title, keywords, and abstract to make a clear decision. In cases where the two reviewers disagreed regarding the eligibility of an article for inclusion, a third reviewer (ZG) was consulted. From the original 214 articles identified via the electronic database search, 23 articles were eligible for inclusion. Nine additional articles were identified by reviewing the bibliographies of the 23 articles. In total, 32 articles were eligible and included in this review (Fig. 1). All included studies relied on self-report. Many of these studies included strong controls for confounders, but due to the early stage of research surrounding safer smoking and harm reduction, all studies fitting inclusion criteria were included regardless of methodological rigor. Due to the early stage of this topic, the authors did not conduct a formal assessment of methodological quality as all included studies were observational and represent low-quality formative evidence. Nonetheless, methodological limitations are reported in the text where relevant.

### Data extraction and analysis

The first and second authors (AT and CA) extracted the following study-level data from the 32 eligible studies using a data collection spreadsheet that included the following domains: Authors, Title, Location/Setting, Participant Characteristics (e.g., people who use drugs or harm reduction organization staff, gender, age group), Study Type (qualitative, quantitative, mixed methods), Main Substance of Focus (e.g., crack cocaine, heroin, any illegal substance), and Study Results.

The 32 articles in this review were then organized using a narrative synthesis approach [36]. Thematic analysis was used in the process of narrative synthesis to develop codes and themes based on the selected studies [37]. The first and second authors (AT and CA) developed the initial set of codes. Codes were then discussed with all coauthors and any recommended changes were discussed and revised until full agreement was reached. The first and second authors then applied the codes to all of 32 studies. After completing the thematic analysis, the codes were then collapsed into five overarching themes. The relevant themes from each study were then extracted and added to the data collection spreadsheet. The Authors, Title, Location, Participant Characteristics, Study Design, Main Substance of Focus, Key Findings, and Overarching Themes for each study are presented in Table 1.

**Table 1:** Summary table of included studies (*N* = 32)

Authors	Title	Location	Participant characteristics	Study design	Substance of focus	Key finding(s)	Theme(s)
Bardwell et al. [39]	Hoots and harm reduction: a qualitative study identifying gaps in overdose prevention among women who smoke drugs	Canada	People who smoke drugs (*n* = 32)	Qualitative cross-sectional study: in-depth interviews	Any substance smoked	Smoking was most common method of drug use (*n* = 29), which was preferred due to negative views of other methods (i.e., injection), how long the high lasted when smoked, bad veins, and limited economic resources Smoking was thought of as harm reduction in and of itself; injection was perceived as having a higher risk of overdose, although some participants were concerned about the risk of overdosing from smoking Participants adapted to overdose risk by smoking smaller amounts more frequently Having a women-only consumption site was seen as beneficial Sharing drugs while smoking was common, and was seen as a method of social cohesion, although others were wary about sharing due to concerns of others stealing their drugs	Smoking as a form of harm reduction
Bourque et al. [67]	Supervised inhalation is an important part of supervised consumption services	Canada	People who smoke drugs (*n* = 654)	Mixed methods: administrative data and cross-sectional study	Any substance smoked	Indoor drug smoking was found as a need for the population; thus, services have been heavily utilized People smoking drugs often do so in groups	Delivery and utilization of safer smoking services
Boyd et al. [45]	Opportunities to learn to barriers to change: crack cocaine use in the Downtown Eastside of Vancouver	Canada	People who smoke drugs (*n* = 27)	Qualitative cross-sectional study: in-depth interviews	Crack	Many participants shared equipment as it was common practice to smoke in small groups in public, thus needing to be vigilant to avoid police harassment Mouthpieces were often used to mitigate sharing risk A lot of what participants knew about harm reduction and smoking was learned from watching others smoke crack Many participants said that there was a need for new paraphernalia to be in circulation Barriers to changing behavior included poor experiences with materials, lack of understanding of risks, and where crack fit in in their lives	Sharing of smoking materials Delivery and utilization of safer smoking services
Bungay et al. [46]	Women’s health and use of crack cocaine in context: Structural and ‘everyday’ violence	Canada	People who use drugs (*n* = 126)	Mixed methods cross-sectional study: survey, participant observations, informal interviews, in-depth interviews	Crack	Participants reported frequently cutting themselves on broken pipes Mouthpieces were preferred as a method to prevent disease transmission, although women were often unable to find one when needed Supplies were expensive to purchase when they were unable to get them from HR agencies Women felt forced to share, especially with men due to fear of violence Police harassment and confiscation of pipes were common, forcing women to share	Sharing of smoking materials
Cheng et al. [47]	Crack Pipe Sharing Among Street-Involved Youth in a Canadian Setting	Canada	People who use drugs (*n* = 567)	Quantitative prospective cohort study: secondary analysis of follow-up survey data	Crack	88% reported sharing pipes during study follow-up period White ethnicity (aOR = 1.34), homelessness (aOR = 1.87), regular employment (aOR = 1.53), daily crack smoking (aOR = 1.37) or crystal methamphetamine use (aOR = 2.04), encounters with police (aOR = 1.42), difficulty accessing pipes (aOR = 1.58) and having unprotected sex (aOR = 1.95) all associated with crack pipe sharing	Sharing of smoking materials
Collins et al. [48]	Potential uptake and correlates of willingness to use a supervised smoking facility for noninjection illicit drug use	Canada	People who inject drugs (*n* = 443)	Quantitative cross-sectional study: secondary analysis of baseline (demographics) and follow-up (drug use behavior) survey data from prospective cohort study	Any substance smoked	Factors associated with willingness to use safer smoking facility: Living in an HIV epicenter (aOR = 1.85), working in the sex trade (aOR = 2.24), and sharing crack pipes (aOR = 1.64)	Sharing of smoking materials Delivery and utilization of safer smoking services
Cortina et al. [66]	Willingness to use an in-hospital supervised inhalation room among people who smoke crack cocaine in Vancouver, Canada	Canada	People who use drugs (*n* = 539)	Quantitative cross-sectional study: secondary analysis of follow-up survey data and serological HIV and HCV testing from two prospective cohort studies	Crack	59.4% of participants said that they were willing to use an in-hospital safe inhalation site Factors associated with willingness: age (aOR = 0.98), daily noninjection crack use (aOR = 1.63)/binge noninjection crack use (aOR = 1.47), difficulty finding new pipes (aOR = 0.51), and ever using drugs in a hospital (aOR = 1.89) HIV positive serostatus = 48% (*n* = 261); HCV serostatus not reported	Delivery and utilization of safer smoking services
Domanico et al. [63]	Implementation of Harm Reduction Toward Crack Users in Brazil: Barriers and Achievements	Brazil	People who smoke drugs (*n* = 30)	Qualitative cross-sectional program evaluation, in-depth interviews	Crack	Funding, high staff turnover and police harassment/violence turnover were a barrier to implementation Engagement of peers was key to success Program participants felt that by having clean supplies distributed by peers, they were able to use more safely	Sharing of smoking materials Delivery and utilization of safer smoking services
Elkhalifa et al. [49]	Combining respondent-driven sampling with a community-based participatory action study of people who smoke drugs in two cities in British Columbia, Canada	Canada	People who smoke drugs (*n* = 149)	Quantitative cross-sectional study: surveys and social network analysis	Any substance smoked	Rural participants more likely to get pipes from stores (29%), peers (24%) Urban participants more likely to get from outreach organizations (89%) More sharing in rural area reported (75% vs. 36% in urban)	Sharing of smoking materials
Frankeberger et al. [50]	Safer Crack Kits and Smoking Practices: Effectiveness of a Harm Reduction Intervention among Active Crack Users in Mexico City	Mexico	People who smoke drugs: Baseline (*n* = 58) Follow-up (*n* = 35)	Quantitative pre-post, single-cohort evaluation of a pilot safer smoking intervention: surveys at baseline and 3-months post-intervention	Crack	At baseline, use of broken pipes/alternative materials were reported frequently (31% said they always used broken pipes) 20% of participants reported at baseline that they shared pipes Overall, respondents showed an increase in use of safer smoking materials Pyrex pipe always or almost always use went up significantly from 7.0 to 27.3% (*p* = .002) There was a significant increase in never/almost never alternate material use (67.2–90.9%, *p* = 0.008) Sharing pipes declined significantly (increase in never/almost never sharing 57.9–87.9%, *p* = .038), as well as sharing alternate materials (59.7–12.9%, *p* = 0.002) Those who received a crack kit were significantly likely to always/almost always use a Pyrex pipe (*p* = 0.040)	Sharing of smoking materials Delivery and utilization of safer smoking services Preliminary efficacy of safer smoking services
Handlovsky et al. [51]	The process of safer crack use among women in Vancouver’s Downtown Eastside	Canada	People who smoke drugs (*n* = 27)	Qualitative cross-sectional study: in-depth interviews	Crack	Establishing safe place for women to smoke was crucial, as many had experienced violence Women were able to engage each other in safer use practices when smoking, e.g., not sharing Sharing pipes were associated with contracting infections and other negative experiences In order to cares for themselves and others, women emphasized the need for safe use equipment, the main barriers being lack of resources and limited hours programs were open	Sharing of smoking materials Delivery and utilization of safer smoking services
Hunter et al. [52]	Reducing widespread pipe sharing and risky sex among crystal methamphetamine smokers in Toronto: do safer smoking kits have a potential role to play?	Canada	People who smoke drugs (*n* = 32)	Qualitative cross-sectional study: focus groups	Methamphetamine	Pipe sharing very common among people who smoke methamphetamine The group that most desired free pipes were homeless youth who did not have the ability to purchase them themselves Gay men and partiers would take free kits if they were conveniently offered; otherwise, they would buy their own pipes Participants doubted that dissemination of kits would reduce pipe sharing as the social aspect of smoking is important	Sharing of smoking materials
Ivsins et al. [53]	Crack pipe sharing in context: How sociostructural factors shape risk practices among noninjection drug users	Canada	People who smoke drugs: Study 1 (*n* = 13), Study 2 (*n* = 31)	Qualitative cross-sectional study: secondary analysis of in-depth interviews data from two studies	Crack	Norms have developed wherein crack smokers share pipes between friends and intimate partners Stigma attached to sharing pipes with strangers Sharing seen as social bonding experience Economic motivations for sharing pipes: you loan someone your pipe; you get a hit off of their rock or build a push Social norms associated with pipe sharing	Sharing of smoking materials
Ivsins et al. [64]	Uptake, benefits of and barriers to safer crack use kit (SCUK) distribution programmes in Victoria, Canada—a qualitative exploration	Canada	People who smoke drugs (*n* = 31)	Mixed methods cross-sectional study; survey and in-depth interviews	Crack	Health benefits from SCUK: preventing infectious disease diagnosis, reducing chances of cutting lips on broken pipes Economic benefits of SCUK: saving money by not having to buy from stores, don't have to take time away from work to go buy pipes Social benefits of SCUK: less crime/stealing pipes, less arguments/violence over pipes Barriers to SCUK: limited hours of distribution, fear of police harassment/violence/breaking pipes	Sharing of smoking materials Delivery and utilization of safer smoking services
Jozaghi et al. [54]	Peer-engagement and its role in reducing the risky behavior among crack and methamphetamine smokers of the Downtown Eastside community of Vancouver, Canada	Canada	People who smoke drugs (*n* = 20)	Qualitative cross-sectional study: in-depth interviews	Any substance smoked	There was a scarcity of high-quality materials until recently High prices of pipes was a major barrier, leading participants to share materials Having a safe use site was vital to mitigate risk of violence Peer work was crucial to engaging participants	Sharing of smoking materials Delivery and utilization of safer smoking services
Leonard et al. [55]	The Urgent Need to Respond to HIV- and HCV-Related Risk Practices among Youth in Ottawa Who Smoke Crack	Canada	People who use drugs (*n* = 97)	Quantitative cross-sectional study: surveys and blood test for HIV and HCV	Crack	61% of women and 49% of men had experienced a nonfatal OD; crack was involved in these OD's 21% of times for women and 1% for men Crack smoking injuries had occurred for 42% of women and 26% of men 57% of women and 56% of men had smoked crack in public 76% of women and 59% of men used glass stems to smoke Other recent materials used to smoke were soda cans (11% of women; 19% of men), inhalers (11% of women; 4% of men) and a metal pipe (4% of men) The majority of participants had never used a mouthpiece (54% of women; 62% of men), the main reasons being the material had a poor taste or feel and that it was challenging to put the mouthpiece on the pipe Use of brass screens was more prevalent than mouthpieces Using a previously smoked out of pipe was common (61% of women; 55% of men recently) Reasons for sharing included lack of resources and fear of police harassment 60% of women and 63% of men actually took safe smoking supplies from a health agency in the last 6 months, however 70% of women and 52% of men reported that they had had challenges accessing these programs at least once Sixty-two finger-prick blood samples were tested for HIV; none was positive Seventy-three finger-prick blood samples were tested for HCV; 15% of women providing samples tested positive for HCV (95%CI: 4.0, 36.0) as did 15% of men (95%CI: 7.3, 26.7)	Sharing of smoking materials Delivery and utilization of safer smoking services
Leonard et al. [56]	"I inject less as I have easier access to pipes": injecting, and sharing of crack smoking materials, decline as safer crack smoking resources are distributed	Canada	People who smoke drugs (*n* = 550): Pre-intervention (*n* = 112), 1-month post-intervention (*n* = 114), 6-month post-intervention (*n* = 157), 12-month post-intervention (*n* = 167)	Quantitative pre-post, evaluation of a pilot safer crack smoking intervention: repeated cross-sectional surveys at 6-moths pre-intervention and 1-, 6- and 12-months post-intervention	Crack	After 1 month, 80% of participants had accessed safer smoking initiative Injecting prior to interview decreased over course of study Majority of participants reported rates of injecting had not changed, but a large proportion of participants reported that they had decreased injecting Majority reported level of engagement with smoking crack had not changed Quarter of participants said that they were smoking more since there was sterile equipment available "Modest downward trend" in sharing across all phases, but significant decrease in frequency of sharing, including post-implementation	Sharing of smoking materials Delivery and utilization of safer smoking services Preliminary efficacy of safer smoking services
Malchy et al. [65]	Do Crack Smoking Practices Change With the Introduction of Safer Crack Kits?	Canada	People who smoke drugs: Pre-intervention (*n* = 206), 1-year post-intervention (*n* = 150)	Quantitative pre-post, evaluation of a pilot safer crack smoking intervention: repeated cross-sectional surveys at pre-intervention and 1-year post-intervention	Crack	Stems and pipes used by almost all participants who received a kit (> 98%)	Delivery and utilization of safer smoking services
Malchy et al. [57]	Documenting practices and perceptions of 'safer' crack use: a Canadian pilot study	Canada	People who smoke drugs (*n* = 97)	Quantitative cross-sectional study: survey	Crack	Most people said they could find crack pipes if they needed them (64%) 80% of participants shared their mouthpieces or pipes People who shared were more likely than those who did not share to sell drugs for sex (56%), experiencing burns (79%), lesions (61%), have a pipe explode (66%), and use broken pipes (87%)	Sharing of smoking materials
McNeil et al. [44]	"We need somewhere to smoke crack": An ethnographic study of an unsanctioned safer smoking room in Vancouver, Canada	Canada	People who smoke drugs (*n* = 23)	Qualitative cross- sectional study: ethnographic observations and in-depth interviews	Crack	Poverty/homelessness restricted access to places where people could smoke crack, especially because preference was to smoke at home Frustrated that safe smoking not incorporated into consumption spaces PWSC highly stigmatized Smoking in public exposed PWSC to violence Pipe sharing was common All participants said that their desire to use SSR was motivated out of wanting to minimize exposure to social violence SSR promoted adoption of risk reduction practices	Smoking as a form of harm reduction Sharing of smoking materials Delivery and utilization of safer smoking services
Parent et al. [38]	Examining prevalence and correlates of smoking opioids in British Columbia: opioids are more often smoked than injected	Canada	People who use drugs (*n* = 369)	Quantitative cross-sectional study: secondary analysis of one-time survey data from a repeated measures monitoring survey	Opioids	Associated odds of smoking opioids include living in a small urban/rural area (aOR 2.41), being a woman (aOR 1.84), under 30 (aOR 5.41), between 30 and 39 years of age (aOR 2.77), using drugs alone (aOR 2.98), and having naloxone (aOR 2.01)	Smoking as a form of harm reduction
Persaud et al. [40]	Controlling Chaos: The Perceptions of Long-Term Crack Cocaine Users in Vancouver, British Columbia, Canada	Canada	People who smoke drugs (*n* = 31)	Qualitative cross-sectional study: focus groups	Crack	Smoking crack allowed participants to exert control over their lives Majority of participants smoked crack in addition to or as a replacement for injecting crack Smoking allowed users to not constantly think about using, compared with injection Participants felt that smoking was safer than injecting Participants felt there was a lack of safe spaces for them to smoke, often necessitating sharing of materials; most preferred to smoke inside for fear of being assaulted Participants felt supervised inhalation site as most helpful to HR	Smoking as a form of harm reduction Sharing of smoking materials Delivery and utilization of safer smoking services
Pizzey et al. [42]	Distributing foil from needle and syringe programs (NSPs) to promote transitions from heroin injecting to chasing: An evaluation	England	People who use drugs (n = 320)	Quantitative cross-sectional evaluation of safer consumption intervention: post-implementation survey and administrative data	Heroin	-Women were more likely to take foils when offered as compared to men (62.3% vs. 44.6%) -Visits to the service programs increased on average by 32.5% after foils were introduced -Several new non injectors started visiting the programs -All participants at the pilot site agreed that having the foils provided was beneficial -Some people who initially refused foil went to use at later date -Providing foils was beneficial to social networks, e.g., participants brought home and encouraged partners to use it/avoid injecting	Smoking as a form of harm reduction Sharing of smoking materials Delivery and utilization of safer smoking services Preliminary efficacy of safer smoking services
Poliquin et al. [41]	Understanding experiences of and rationales for sharing crack smoking equipment: A qualitative study with persons who smoke crack in Montréal	Canada	People who use drugs (*n* = 26)	Qualitative cross-sectional study: focus groups	Crack	Many injectors had transitioned to smoking for health reasons/being tired of injecting All participants were aware of agencies or businesses they could get or buy smoking equipment from; however, some participants thought there was not enough availability in hotspots Materials were still seen as easy to get Pipes were shared for a variety of reason: not wanting to appear as a "drug addict" and have materials on hand, to maintain social bonds, feeling pressured to share, to build a push/save money, or when intoxicated Perceived risk varied; some participants kept their pipes to themselves out of germaphobia, while others were skeptical about the risk for infection from sharing Using mouthpieces was common among participants, as well as only sharing with people the participants knew, as well as keeping extra pipes to give out to others (prevention strategies)	Smoking as a form of harm reduction Sharing of smoking materials Delivery and utilization of safer smoking services
Prangnell et al. [68]	Declining rates of health problems associated with crack smoking during the expansion of crack pipe distribution in Vancouver, Canada	Canada	People who inject drugs (*n* = 1718)	Quantitative prospective cohort study: secondary analysis of survey data and HIV and HCV blood test data from two prospective cohort studies; collected at enrollment and every 6 months thereafter	Crack	Proportion of study participants reporting health issues related to smoking crack declined by 18.5% over the study period Participants accessing crack pipes only through a health service (as compared to friends, bodegas) increased significantly (7.2–62.3%) Obtaining pipes through health service associated with decreased health issues 41% (*n* = 698) of the same were HIV positive; HCV results were not reported	Delivery and utilization of safer smoking services Preliminary efficacy of safer smoking services
Rigoni et al. [58]	From opiates to methamphetamine: building new harm reduction responses in Jakarta, Indonesia	Indonesia	Staff and people who use drugs: Survey of management (n not reported), Staff interviews (*n* = 8), Service user interviews (*n* = 2), Service user focus group (*n* = 10)	Mixed methods cross-sectional study: review of contextual and program documents, survey of management, field observations, in-depth interviews with service providers and service users, a focus group with service users	Any substance	Involving peers was a vital step to reaching stimulant users Most people said that they shared materials, thus program staff adapted distributed materials to include silicone mouth pieces to reduce risk	Sharing of smoking materials Delivery and utilization of safer smoking services Preliminary efficacy of safer smoking services
Shannon et al. [35]	Potential community and public health impacts of medically supervised safer smoking facilities for crack cocaine users	Canada	People who smoke drugs (*n* = 437)	Quantitative cross-sectional study: survey	Crack	Factors associated with willingness to use safer smoking facility: current IDU (aOR 1.72), equipment confiscated/broken by police (aOR 1.96), crack bingeing (aOR 2.16), smoking crack in public (aOR 2.48), borrowing crack pipes (aOR 2.5), inhaling Brillo/getting burned due to rushed crack use (aOR 4.37)	Sharing of smoking materials Delivery and utilization of safer smoking services
Stöver et al. [43]	SMOKE IT! Promoting a change of opiate consumption pattern-from injecting to inhaling	Germany	People who use drugs: T1 (*n* = 165) T2 (*n* = 141) T3 (*n* = 89)	Quantitative single-cohort pre-post evaluation of a safer smoking intervention: survey at baseline (T1), post-intervention (T2), and 30-days after T2 (T3)	Any substance smoked	65.3% of the participants used the foils rather than injecting 58.9% of participants said that they preferred smoking with the foils over injecting because it was healthier, 35.7% because of the reduced risk of HIV/HCV, 33.9% to avoid OD 87.6% of participants continued to use the foils in the third study period	Smoking as a form of harm reduction Delivery and utilization of safer smoking services Preliminary efficacy of safer smoking services
Strike et al. [59]	Education and equipment for people who smoke crack cocaine in Canada: progress and limits	Canada	Staff (*n* = 80)	Quantitative cross-sectional study: survey data and secondary analysis of a prior survey	Crack	Majority of programs reported that they did provide education sharing risk reduction for smoking (76%), including on risks from improvised equipment (75%), and how to use safer smoking equipment (72%) 64% of program managers reported that they distributed safer smoking materials, including pipes (96%), mouthpieces (94%), screens (94%), and push sticks (92%) For those that were not able to distribute these materials, the most common reasons were funding (32%) and lack of demand (25%)	Sharing of smoking materials Delivery and utilization of safer smoking services Preliminary efficacy of safer smoking services
Ti et al. [60]	Difficulty accessing crack pipes and crack pipe sharing among people who use drugs in Vancouver, Canada	Canada	People who use drugs (*n* = 503)	Quantitative cross-sectional study: secondary analysis of follow-up survey data and serological HIV testing from two prospective cohort studies	Crack	47.3% of participants shared a pipe during the past 6 months Factors associated with sharing a pipe were: having acquired a mouthpiece (aOR 1.91), challenges accessing pipes (aOR 2.19) and binging noninjection drugs (aOR 1.39)	Sharing of smoking materials
Ti et al. [61]	Factors associated with difficulty accessing crack cocaine pipes in a Canadian setting	Canada	People who use drugs (*n* = 914)	Quantitative prospective study: secondary analysis of follow-up survey data and serological HIV and HCV testing from two prospective cohort studies	Any substance	Characteristics of people who had difficulty accessing pipes included doing sex work (aOR 1.57), having shared a crack pipe (aOR 1.69), having police be present where drugs are bought or used (aOR 1.47), difficulty accessing services (aOR 1.74) and reporting health problems (aOR 1.37) HIV positivity = 54% (*n* = 498); HCV positivity not reported	Sharing of smoking materials Delivery and utilization of safer smoking services
Voon et al. [62]	Risky and rushed public crack cocaine smoking: The potential for supervised inhalation facilities	Canada	People who use drugs (*n* = 1085)	Quantitative prospective cohort study: secondary analysis of follow-up survey data and serological HIV and HCV testing from two prospective cohort studies	Any substance	Factors associated with public crack use: younger age (aOR 1.03), homelessness (aOR 3.48), dealing drugs (aOR 1.59), daily or more crack smoking (aOR 2.69), sharing a crack pipe (aOR 1.98), public injection use (aOR 5.42), noticing police presence (aOR 1.3), and a history of incarceration (aOR1.47) Factors associated with rushed public crack use: younger age (aOR 1.02), homelessness (aOR 1.23), dealing drugs (aOR 1.39), smoking crack daily or more (aOR 1.48), and sharing crack pipes (aOR 1.44) At baseline, 46% (*n* = 496) of the sample had an HIV positive serostatus; HCV serostatus was not reported	Sharing of smoking materials

*aOR* Adjusted odds ratio, *SCUK* safe crack use kit, *OD* overdose, *SSR* safe smoking room, *PWSC* people who smoke crack, *HR* harm reduction, *IDU* injection drug use, *HIV* human immunodeficiency virus

## Results

### Study characteristics

Figure 1 depicts the study selection process. In total, 32 articles were eligible and included in this review (Table 1). All 32 articles were observational, of which, 18 employed quantitative methods (14 surveys; 5 serology, 1 secondary data collection), ten employed qualitative methods (six in-depth interviews; two focus groups), and four utilized mixed methods. Overall, 25 of the studies were one-time cross-sectional studies, and seven were longitudinal studies.

The studies were published between 2005 and 2021. All included studies were conducted outside of the U.S., with the majority coming from Canada (*n* = 27) and 1 each coming from Brazil, England, Germany, Indonesia, and Mexico.

Overall, 30 studies exclusively included people who use drugs as participants (*n* = 11 people who use drugs; generally, *n* = 17 people who smoke drugs, *n* = 2 people who inject drugs). One study included harm reduction program staff serving people who use drugs, and one study included staff and people who use drugs. Several studies examined specific substance use patterns among people who use drugs, including the use of crack cocaine, methamphetamine or multiple substances. The majority of the studies (*n* = 20) focused on crack use, six on any substance smoked, three on any illegal drug used, two on methamphetamine use, and one each on methamphetamine, opioids, and heroin.

### Overarching themes

#### Smoking as a form of harm reduction

One quantitative study with people who use drugs examined both the social and behavioral factors associated with smoking opioids [38]. The researchers found that when adjusting for smoking opioids, participants who used methamphetamine had 6 times higher odds of smoking opioids (adjusted Odds Ratio (aOR) = 6.48; 95% confidence interval (CI)  3.51–11.96, *p* < 0.01) than those who did not use methamphetamine. Other factors associated with the increased odds of smoking opioids include living in a small urban/rural area (ref = median/large urban area; aOR = 2.41, 95% CI  1.27–4.58, *p* = 0.01), being a woman (ref = man; aOR 1.84, 95% CI 1.03–3.30, *p* = 0.04), being under age 30 (ref = 50 and over; aOR = 5.41, 95% CI  2.19–13.40, *p* < 0.01), between 30 and 39 years of age (ref = 50 and over; aOR = 2.77, 95% CI  1.33–5.78, *p* = 0.01), using drugs alone yes vs. no; aOR 2.98, 95% CI  1.30–6.83, *p* = 0.01), and having naloxone (yes vs. no; aOR = 2.01, 95% CI  1.08–3.72, *p* = 0.03) [38].

Five qualitative studies [38–41] and two quantitative studies [42, 43] examined how smoking as opposed to injecting substances is a form of harm reduction. Specifically, in two qualitative studies [40, 44], participants who smoked drugs as opposed to injected drugs reported feeling more in control of their lives and able to take care of themselves and their needs, such as their health and housing. Further, in one of the qualitative study with people who smoke drugs [40], a participant explicitly noted that she felt more socially and fiscally stable since ceasing injecting drug use. Participants in another qualitative study with women who smoke drugs [39] expressed a similar preference for smoking as opposed to other modalities. When describing their partiality to smoking over injecting, across studies, many participants reported a fear of needles/syringes and acknowledged that although there was still some risk of overdose when smoking drugs, smoking carried less overdose risk than injecting drugs. In addition to acknowledging the reduced risk for overdose with smoking as opposed to injecting, people who use drugs in one qualitative study [41] described smoking as a way to reduce HIV and HCV acquisition risk, compared to injecting. Comparably, 58.9% of the 112 people who use drugs who participated in one quantitative study [43] indicated that they preferred smoking with foils (heating heroin on a piece of aluminum foil and inhaling the vapor through a straw) over injecting as they believed it to be healthier. Additionally, 35.7% of people who use drugs in the same study reported that smoking drugs (instead of injecting) reduced their risk of HIV or HCV, and 33.9% reported that smoking helped to reduce their risk of overdose.

#### Sharing of smoking materials

Although participants in many studies reported that smoking carried fewer health risks than injecting drugs, many people who use drugs in the included studies reported sharing smoking materials, which can increase individuals’ risk of disease acquisition and transmission. Indeed, 23 studies included data on the prevalence of and rationale for the sharing of smoking materials [35, 40–42, 44–62].

Prevalence of sharing smoking materials was reported in five studies. In one quantitative study with people who use drugs, 88% of the 567 participants reported sharing crack smoking materials [47]. Another quantitative study with 149 people who smoke drugs found that over half (56.38%) of their participants had loaned, borrowed or shared pipes [49]. Similarly, in a quantitative study with people who use drugs, 47.3% of the 503 participants had shared a crack pipe in the last 6 months [60]. Just under half (48.57%) of the 1085 people who use drugs in a quantitative study reported sharing materials [62]. One study found the sharing of materials differed somewhat by gender such that 61% of women and 55% of men in the study reported sharing smoking materials in the 6 months prior to participating in the study [55].

Several studies with people who use drugs and people who smoke drugs identified a myriad of reasons for why people who use drugs reported shared smoking materials. Participants in one qualitative study with people who use drugs [41] and two qualitative studies with people who smoke drugs [45, 53] provided economic reasons for sharing materials, such as building a “push” of crack resin (i.e., allowing small amounts of resin from previous crack smoking sessions to accumulate to be smoked again). Participants in another qualitative study with people who smoke drugs reported concerns about the high price of pipes [49]. Further, participants in one qualitative [54] and one quantitative [56] with people who smoke drugs spoke of challenges in finding new materials to use in the context of limited resources. Additionally, participants in one qualitative [41] and one mixed methods [46] study with people who use drugs, and a qualitative study with people who smoke drugs [45] also reported that they do not always carry their own pipes.

Sharing for social reasons was also commonly reported. Specifically, across studies using qualitative approaches [39, 41, 44, 51–53] people who use drugs and people who smoke drugs reported that crack and methamphetamine smoking are viewed as social activities and beneficial to positive group dynamics such as protection of others within the group among communities of people who use drugs.

Two quantitative, one qualitative and one mixed methods study examined factors associated with pipe sharing [48, 50, 63, 64]. One study [47] used logistic regression and found that the following sociodemographic factors were significantly and positively associated with pipe sharing: homelessness; (yes vs. no; aOR = 1.87, 95% CI  1.43–2.44, *p* < 0.001), regular employment; (yes vs. no; aOR = 1.53, 95% CI  1.15–2.04, *p* = 0.003), daily crack smoking; (yes vs. no; aOR = 1.37, 95% CI 1.01–1.85, *p* = 0.043), crystal meth use; (yes vs. no; aOR = 2.04, 95% CI  1.11–3.75, *p* = 0.022), encounters with police; (yes vs. no; aOR = 1.42, 95% CI  1.01–1.99, *p* = 0.043), having unprotected sex; (yes vs. no; aOR = 1.95, 95% CI  1.47–2.58, *p* < 0.001). Another study that employed logistic regression (62) found that sharing a crack pipe was significantly associated with the increased odds of smoking crack in public; (yes vs. no; OR = 1.68, 95% CI  1.26–2.25, *p* < 0.001) reported sharing pipes. Two additional studies [49, 57] examined global differences in the sociodemographic characteristics of those who shared pipes, with one study [49], finding that a significantly higher proportion of people living in a rural area as opposed to a major urban area shared pipes (*p* < 0.01). The other study found that compared to those who did not share pipes, a higher proportion of those who reported sharing pipes also reported selling drugs for sex, experienced burn or lesions, had a pipe explode, and used broken pipes [57]. Additionally, in three quantitative studies with people who use drugs [47, 60, 61] challenges accessing pipes was significantly and positively associated with the increased odds of sharing pipes with others (yes vs. no; aOR = 1.58, 95% CI 1.13–2.20; *p* = 0.007 [42]; aOR = 2.19, 95% CI  1.42—3.37; *p* < 0.01 [60]; aOR = 1.74, 95% CI  1.31–2.32, *p* < 0.01 [61]).

#### Delivery and utilization of safer smoking services

##### Utilization of smoking services

**Fig. 2:**
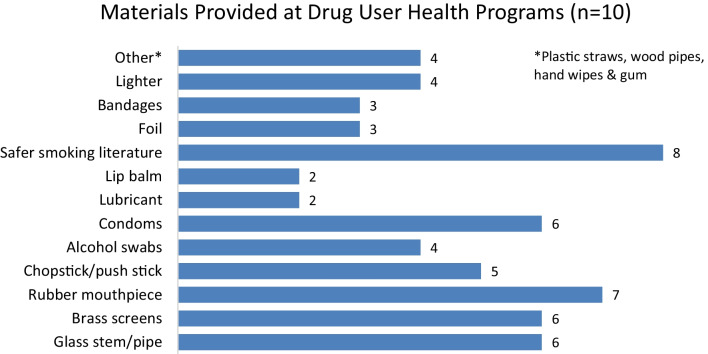
Type of materials provided at drug user health programs

In exploring the utilization of smoking services, the harm reduction programs featured across ten of the included studies were described as providing a variety of materials to their clients [35, 42, 43, 45, 50, 56, 58, 63–65]. Specifically, as shown in Fig. 2, harm reduction organizations provided glass pipes, rubber mouthpieces, brass tobacco screens, wooden push sticks, condoms and descriptive literature.^1^

Eight of the studies included in this review evaluated safer drug smoking initiatives [42, 43, 50, 56, 63, 65]. In a quantitative study with 80 program staff evaluating drug user health programs across Canada, participants reported that the majority of programs provided education on risk reduction for smoking (76%), including education on risks from improvised equipment (75%), and how to use safer smoking equipment (72%) [59]. Several studies also described how safer smoking programs were modified over time to meet the needs of people who use drugs. In one mixed methods study with eight harm reduction staff [58], upon receiving feedback that most participants shared smoking materials, harm reduction staff modified the materials they distributed to people who use drugs by including a mouthpiece in their safer smoking kits.

##### Access to and feasibility and acceptability of safer smoking services

Overall, several studies examined the anticipated and actual utilization of safer smoking services by people who use drugs with (*n* = 443) [48] and without (*n* = 437) [35] experiences accessing these services. Indeed, willingness to use safer smoking materials or safer smoking facilities’s ranged from 27.99% [43] to 69% [35] across studies of people who use drugs. Additionally, in both previously mentioned quantitative studies with people who use drugs[35, 48], found that compared to those who did not share materials, those who shared pipes had significantly greater odds of reporting a willingness to use safer smoking facilities (aOR = 1.64, 95% CI 1.02–2.64, *p* = 0.042 [48], aOR = 2.5, 95% CI 1.86–3.40, *p* = 0.006 [35]). Further, across three studies [35, 48, 66], additional factors associated with willingness to use a safer smoking facility included living in an HIV epicenter (yes vs. no; aOR = 1.85; 95% CI 1.14–2.97, *p* = 0.011), working in the sex trade (yes vs. no; aOR = 2.24, 95% CI  1.32–3.80, *p* = 0.003) [48], daily noninjection crack use (yes vs. no; aOR = 1.63, 95% CI  1.08–2.48, *p* = 0.021), binging crack (yes vs. no; aOR = 2.16, 95% CI  1.39–3.12, *p* = 0.014), ever using drugs in a hospital (yes vs. no; aOR = 1.89, 95% CI  1.31–2.73, *p* < 0.001) [66], current injection drug use (yes vs. no; aOR = 1.72, 95% CI  1.09–2.70, *p* = 0.019), having equipment confiscated or broken by the police (yes vs. no; aOR = 1.96; 95% CI  1.24–2.85, *p* = 0.003), smoking crack in public (yes vs. no; aOR = 2.48, 95% CI  1.65–3.27, *p* = 0.002), and inhaling Brillo/getting burned due to rushed crack use (yes vs. no; aOR = 4.37, 95% CI  2.71–8.64, *p* < 0.001) [35]. In one quantitative study with people who use drugs [66], difficulty finding new crack pipes was negatively associated with willingness to use a safer smoking facility (yes vs. no; aOR = 0.51; 95% CI  0.30–0.86, *p* = 0.013).

Notably, ten studies [42–44, 50, 56, 63–65, 67, 68] found that people who use drugs were already utilizing safer smoking materials and programs, some at very high levels of utilization. Specifically, one quantitative study with people who smoke drugs found that 80% of participants accessed the safer smoking program being evaluated within 1 month of the program opening [56]. Similarly, in a quantitative study with people who smoke drugs, 98% of participants reported using the glass stems and pipes in the safer smoking kits that were distributed at a harm reduction organization in Canada [65].

In 11 of the included studies [40, 41, 44, 45, 51, 54, 55, 59, 61, 63, 64], participants who used drugs reported multiple barriers to accessing safer smoking materials. Across studies, lack of resources was cited as a common barrier to people who use drugs’s ability to access safer smoking harm reduction materials. These resources included lack of funding for programs to give out safer smoking equipment [63], as well as not having enough sterile materials in circulation that participants were able to access [41, 51, 55, 59], fears of harassment by the police and/or violence due to police interaction was another common barrier to accessing safer smoking materials [55, 61, 63, 64]. For example, in one mixed methods study with people who smoke drugs, participants commonly reported having their pipes confiscated by police or taken and immediately broken [64].

In some cases, even when safer smoking materials were being offered in a specific community, people who use drugs could not consistently or easily access them. For example, one qualitative and one mixed methods studies with people who smoke drugs [51, 64] and one quantitative study with people who use drugs [55] found that the limited hours of operations of harm reduction programs were a barrier to accessing safer smoking materials when needed. Other barriers experienced by research participants included a lack of safe spaces in which to smoke [40, 44], poor experiences with smoking materials (e.g., not liking using screens) [45], high staff turnover [63], and a lack of demand from participants for safer smoking materials [59].

Notably, three qualitative and one mixed methods studies found that having peer staff working in harm reduction agencies connect with, and distribute materials to, people who use drugs was crucial to client engagement with services [45, 54, 58, 63]. Further, in one of these studies in which both staff and clients participated, people who use drugs reported that they felt safer using harm reduction services when they were distributed by peers with a history of drug use [63].

## Preliminary efficacy of safer smoking services

Overall, there were six studies that assessed the impact of safer smoking services on health behaviors and wellbeing. Five program evaluation studies found that participants’ use of smoking equipment, sometimes over injecting, increased as materials were provided. A quarter of participants in one quantitative study with people who use drugs reported that they were smoking more since there was sterile equipment made available to them [56]. In another quantitative study with people who smoke drugs [50], the proportion of participants who reported always or almost always using a Pyrex pipe (a preferred material due to the higher durability material compared to regular glass pipes [69] increased significantly from 7.0 to 27.3% (*p* = 0.002). Additionally, in a quantitative study with people who use drugs [42], all participants felt that the single use foils they received were beneficial to have at their harm reduction program. Participants in a quantitative study with people who use drugs provided context as to why participants preferred smoking with single use foils; 58.9% of participants said that they preferred smoking with the foils over injecting because it was healthier, 35.7% because of the reduced risk of HIV/HCV, and 33.9% to avoid overdose [43].

Four studies evaluated interventions to reduce the sharing of smoking materials [50, 56, 58, 59]. In two quantitative studies with people who smoke drugs, the researchers found that providing new pipes to people who use drugs resulted in decreased sharing of smoking equipment over the study period [50, 56]. Additionally, in a quantitative study with people who use drugs, participants who received safer smoking materials not only reported reducing their injection drug use behaviors but also reported bringing back safer smoking materials to their friends and other people in their drug use network [42].

Only one study directly assessed the impact of safer smoking programs on health outcomes. Specifically, one quantitative study with 1718 people who smoke drugs who had received safer crack smoking materials found that participants’ health issues (e.g., burns, sores, coughing blood) related to smoking crack declined by 18.5% over the study period (December 2005–November 2014) [68].

## Discussion

This is the first review, to our knowledge, to synthesize the available literature on safer smoking practices, and safer smoking service delivery and utilization. Findings show that smoking drugs is a popular route of administration among people who use drugs and evidence from this review suggests that expanding access to safer smoking within harm reduction services is crucial to risk mitigation. Within the studies included in this review, most study participants, including people who smoke drugs, peers, and service providers, believed safer smoking services to be a necessary harm reduction intervention, especially when considered in relation to existing safer injection services [39, 40, 42–44, 51, 54, 56, 63, 64, 67, 68]. Further, across studies, people who use drugs reported a high willingness to utilize these services, and in places where services were offered, many studies reported high utilization of safer smoking services. Additionally, although efficacy data were limited, across studies, people who use drugs reported decreasing their injection drug use in favor of smoking, reducing the sharing of smoking equipment, and in some cases improved health outcomes (e.g., decreased burns and cuts). Despite the clear benefits of safer smoking practices, some people who use drugs and service providers reported ongoing barriers to accessing and delivering these services, respectively. Findings underscore the need for ongoing research and structural interventions to increase access to safer smoking programs and reduce drug use related morbidity and mortality.

This is a burgeoning area of research, which we expect to grow and evolve as policies shift, more funding becomes available for the inclusion of safer smoking kits into harm reduction service offerings, and the benefits of these practices become more well known. In fact, since the time that this search was conducted, a new study was published in May 2023 that showed high interest in using safer smoking materials, with participants believing it would reduce their injection use of drugs. As additional studies are published, including those that are based on higher quality evidence, we anticipate a need to update this review in future years [70].

Despite evidence that smoking has benefits over injecting [39, 40, 42–44, 51, 54, 56, 63, 64, 67, 68], across studies, people who use drugs report programs providing safer smoking materials are a minority among harm reduction organizations globally. Ongoing work is needed to incorporate safer smoking materials into the services provided by existing harm reduction organizations. The studies reviewed here provide evidence of the presence of peer workers who are part of these communities as people with lived experience and found peers to be integral in engaging people who use drugs and assisting them with changing their practices. Further outreach to educate people who use drugs about smoking as a harm reduction practice is necessary, including the nuanced benefits and risks associated with it.

In addition to program adaptations, there is also a need for additional research related to safer smoking services. Specifically, the vast majority of studies included in this review focused on crack smoking, demonstrating the need to better understand how people smoke drugs other than crack. Such data are essential to learning how to adapt safer smoking equipment in order to reduce smoking related harms and improve the acceptability of the safer smoking materials provided to people who smoke drugs.

Notably, none of the studies included in this review were based in the U.S. or other countries where smoking is banned. In the U.S. for example, Alaska is the only state that has amended its constitution to remove safer smoking materials from their definitions of ‘paraphernalia’ or protect individuals from criminal charges for possession of safer smoking materials if they were obtained from an authorized harm reduction organization, despite evidence that these types of possession laws can further harm people using substances [71]. These policy shifts have enabled harm reduction organizations in several states to begin disseminating safer smoking materials; however, these programs have yet to be formally evaluated and documented in the literature. As safer smoking services become more widely available in the U.S. and worldwide, it is essential that efforts be made to support community programs in building the infrastructure to rigorously evaluate the impact and efficacy of safer smoking service delivery. High-quality data on the feasibility, acceptability, and efficacy of these programs in U.S. and similar country’s drug use contexts and beyond is necessary to secure sufficient allocation of supportive resources for safer smoking materials delivery in harm reduction, community, and medical settings as well as identify intervention targets aimed at improving access to and utilization of safer smoking services.

Canadian research was the main source for studies included in this review. Canada has been distributing safer smoking materials since the early 2000’s and as such, researchers have had a plethora of material to study. Smoking is the most common route of administration in some provinces of Canada [72], and in response to the increased overdose deaths attributed to smoking opioids, the government took steps to reduce barriers to safer smoking resources by authorizing some safe consumption sites to offer inhalation spaces. Thus, the research coming out of Canada was most relevant to this review.

Finally, although some U.S. states or districts have decriminalized the provision of drug use paraphernalia [73], ongoing policy shifts are necessary to ensure continued access to these essential tools for people who use drugs. Specifically, under current policy, U.S. harm reduction agencies receiving financial assistance from federally funded grants are not able to purchase pipes or stems with those funds [74]. This leaves harm reduction agencies reliant on individual donations or small state or private grants to procure safer smoking material, if they are purchased at all. It is necessary for lawmakers, funders, and the broader community to recognize safer smoking practices and supplies as equally valuable and essential as safer injection practices and supplies given the small but growing evidence of the need for and health-related benefits of these services. Findings from this review underscore the necessity of ensuring that harm reduction services for people who smoke drugs, and the agencies that serve them, be given the same attention and financial support as services designed for people who inject drugs.

### Limitations and strengths

This review has several limitations. All included studies were observational or retrospective and were thus subject to recall bias. Due to the social stigma surrounding substance use, study participants may have underreported some behaviors. Since the included studies had small sample sizes their findings may not be applicable to larger samples or different contexts, such as geographic regions, ethnicities, or genders.

Despite these limitations, this review also has strengths. All 32 studies included in this review are from peer-reviewed academic journals. To the authors’ knowledge, this is the first systematic review examining safer smoking in the harm reduction context and thus, provides synthesized information not previously available in the literature.

## Conclusion

Overall, findings from this systematic review underscore the great need for harm reduction service providers to adapt their services to meet the needs of people who smoke drugs. Service adaptation will require changes in policy and practice to improve the availability and dissemination of safer smoking materials to people who smoke drugs. Consumption sites inclusive of safer smoking are valuable resources that need to be available to support harm reduction activities for people who smoke drugs. Additionally, ongoing high-quality research is needed to better understand how people smoke drugs and the feasibility, acceptability, and efficacy of safer smoking services in the U.S. and globally.

## Data Availability

Not applicable.

## Appendix: Search strategy

**Table:**

SET	Topic	Search terms
1	Safe smoking	Safe* smoking
2		Safer smoking practices
3		People who smoke drugs
4		Pipes
5	Harm reduction	Harm reduction
*A. Set 1–5 were merged with “AND”*		
6	Exclusions	Tobacco
7		Cannabis
8		Marijuana
*B. Set 6–8 were merged with “NOT”*		
*C. Set 1–8 were combined*		
*D. De duplicated*		

## Footnotes

1 Glass stem/pipe- glass tubes used most often to smoke crack or methamphetamine, Brass screens- a filter used to hold a crack rock in place in a stem/pipe, Rubber mouthpiece- barrier used at the end of pipe to protect against cuts, burns, and disease transmission and can be switched out when sharing stems/pipes, Chopstick/push stick- used to pack the filter into place and push filter around to collect residue, Lip balm-to protect against chapped lips due to smoking, Foil-small squares of aluminum foil used most often to smoke opiates, Plastic straws-Used for safer sniffing of drugs.
